# Local Piezoelectric Properties of Doped Biomolecular Crystals

**DOI:** 10.3390/ma14174922

**Published:** 2021-08-30

**Authors:** Andrei Kholkin, Denis Alikin, Vladimir Shur, Shiri Dishon, David Ehre, Igor Lubomirsky

**Affiliations:** 1 School of Natural Sciences and Mathematics, Ural Federal University, 620000 Ekaterinburg, Russia; denis.alikin@urfu.ru (D.A.); vladimir.shur@urfu.ru (V.S.); 2 Department of Physics & CICECO-Aveiro Institute of Materials, University of Aveiro, 3810-193 Aveiro, Portugal; 3 Research School of Chemistry & Applied Biomedical Sciences, National Research Tomsk Polytechnic University, 634050 Tomsk, Russia; 4 Department of Molecular Chemistry and Materials Science, Weizmann Institute of Science, Herzl St 234, Rehovot 7610001, Israel; shiri.dishon@weizmann.ac.il (S.D.); david.ehre@weizmann.ac.il (D.E.); igor.lubomirsky@weizmann.ac.il (I.L.)

**Keywords:** α-glycine, piezoelectricity, doping, piezoresponse force microscopy

## Abstract

Piezoelectricity is the ability of certain crystals to generate mechanical strain proportional to an external electric field. Though many biomolecular crystals contain polar molecules, they are frequently centrosymmetric, signifying that the dipole moments of constituent molecules cancel each other. However, piezoelectricity can be induced by stereospecific doping leading to symmetry reduction. Here, we applied piezoresponse force microscopy (PFM), highly sensitive to local piezoelectricity, to characterize $\left(0\bar{1}0\right)$ faces of a popular biomolecular material, α-glycine, doped with other amino acids such as L-alanine and L-threonine as well as co-doped with both. We show that, while apparent vertical piezoresponse is prone to parasitic electrostatic effects, shear piezoelectric activity is strongly affected by doping. Undoped α-glycine shows no shear piezoelectric response at all. The shear response of the L-alanine doped crystals is much larger than those of the L-threonine doped crystals and co-doped crystals. These observations are rationalized in terms of host–guest molecule interactions.

## 1. Introduction

The amino acid glycine is the simplest building block of various biomaterials and often considered as a symbol of life on our planet. Therefore, it has attracted a considerable amount of attention in different research fields, mostly in materials science, pharmacology, and medicine. Glycine plays a key role in many physiological processes, e.g., in cancer cell metabolism [1], and it is used to treat various diseases, including ischemic stroke, anxiety, insomnia, schizophrenia, benign prostatic hyperplasia, etc. [2,3]. It also serves as a bulking agent in pharmaceutical protein formulations [4]. Though glycine consists of strongly polar molecules, its most frequently used α polymorph is non-polar (when eliminating surface pyroelectric and piezoelectric effects, it originates from the incorporation of water in the surface during the crystal growth [5]). However, its β- [6] and γ-polymorphs [7,8] hold great promise for piezoelectric applications, mainly mechanical energy harvesters. Glycine has a lot of advantages compared to traditional piezoelectric materials, such as PZT and PVDF, due to its biocompatibility and degradability in implantable devices. The way to induce piezoelectric properties in non-polar α-glycine is to dope it with other amino acids that induce a total dipole moment, P (and thus piezoelectricity). This polarity of doped crystals comes from two contributions. Apparently, guest molecules have a different dipole moment than that of the host that it replaces and it thus contributes to P. Also, the asymmetric distortions introduced by the dopant may break the symmetry and force the dipoles of the neighboring host molecules out of compensation, thereby contributing to the overall polarity of the doped crystals as well. This was clearly demonstrated by Meirzadeh et al. [9].

In this work, we applied the piezoresponse force microscopy (PFM) method to study tiny vibrations (of the order of tens of fms) excited on the surfaces of undoped and doped (0.5% wt concentration) α-glycine crystals grown from the solution. We show that it is the shear piezoresponse (rather than the vertical one) that can serve as a reliable tool to study the effect of doping on piezoelectricity.

## 2. Materials and Methods

Mixed crystals of α-glycine (group P_21/n_) were grown by slow evaporation in a clean room environment at 23 °C from aqueous solutions of glycine (99.5+%, Alfa Aesar, Lancashire, United Kingdom) in the presence of 5% wt·wt^−1^ L-alanine (99.2%, Chem-Impex Int’l Inc, Wood Dale, IL, USA), L-threonine (99.0–100.0%, Fisher Scientific, Waltham, MA, USA), or both. The transparent single crystals were washed in water and then dried and heated to 100 °C for 24 h to remove surface polarization induced by the incorporation of water at the surfaces of the crystals. The top {010} face of the pure α-glycine crystals was cleaved. All commercial materials were used as received. The dopant concentrations in the crystals were measured using liquid chromatography mass spectroscopy (LC-MS).

Experiments were conducted on the cleaved crystals stored at ambient conditions. PFM measurements were performed using an NTEGRA Aura scanning probe microscope (NT-MDT, Zelenograd, Russia) and HFLI external lock-in amplifier (Zurich Instruments, Zurich, Switzerland). The measurements were performed in dual-frequency resonance tracking mode. ScanSens HA_NC cantilevers with a 3.5 N/m spring constant and around 140 kHz free flexural resonance were used. The measurements were done in air at about 40–50% humidity. A 2 kHz frequency window and 8 V amplitude AC bias was used for scanning. Calibration of the measured probe tip displacements was made based on the quasi-static measurements of the force–distance curves [10,11]. The measurements were performed on the $\left(0\bar{1}0\right)$ facets of crystals with different doping parameters: undoped α-glycine; α-glycine doped with threonine or alanine; and α-glycine crystals co-doped with threonine or alanine. To increase the signal-to-noise ratio, the measurements were performed with tracking of the resonance frequency using the incorporated dual-frequency resonance tracking (DFRT) mode of the HFLI lock-in amplifier. The obtained values of the response captured in resonance were divided by a quality factor Q and adjusted by a resonance mode shape factor [12,13]. The quality factor of the first flexural contact resonance was about 300, which made it possible to achieve extremely high sensitivity in the measurements and register the signal in the range 10–100 fm/V.

## 3. Results and Discussion

**Figure 1:**
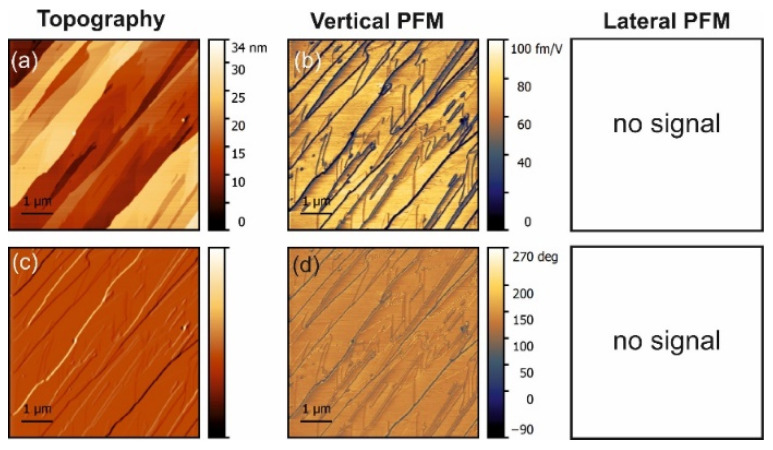
PFM measurements in undoped glycine. (**a**) Topography and (**c**) deflection; (**b**) amplitude and (**d**) phase of the vertical piezoresponse. The lateral PFM was null because no resonance could be excited.

**Figure 2:**
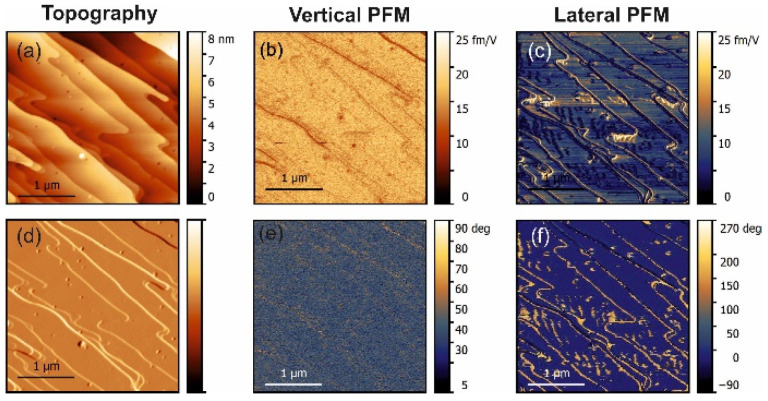
PFM measurements in threonine-doped glycine. (**a**) Topography and (**d**) deflection; (**b**) amplitude and (**e**) phase of the vertical piezoresponse; (**c**) amplitude and (**f**) phase of the lateral piezoresponse.

**Figure 3:**
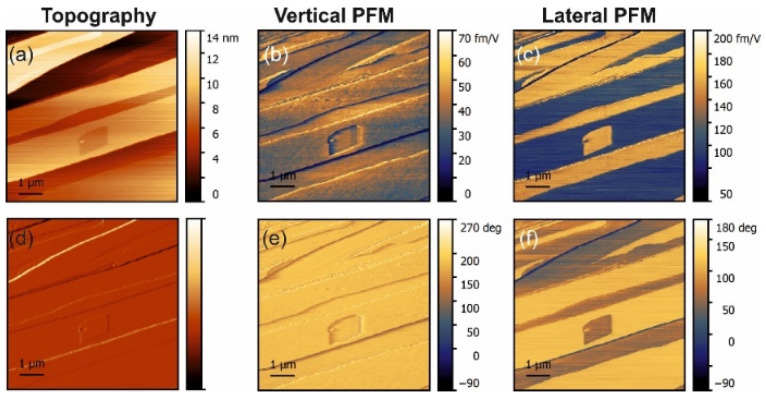
PFM measurements in L-alanine-doped glycine. (**a**) Topography and (**d**) deflection; (**b**) amplitude and (**e**) phase of the vertical piezoresponse; (**c**) amplitude and (**f**) phase of the lateral piezoresponse.

**Figure 4:**
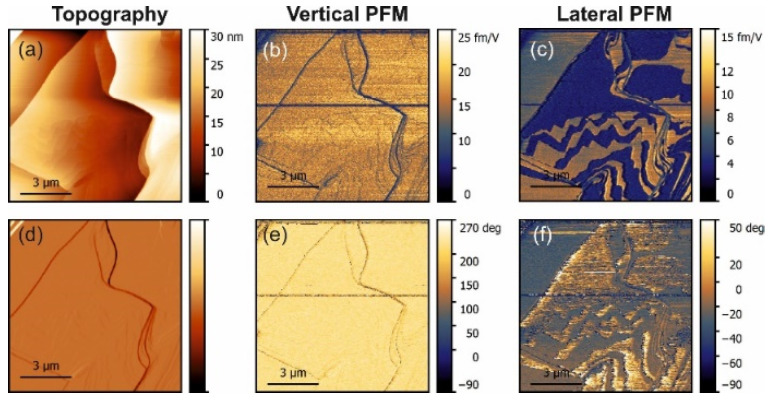
PFM measurements in glycine with L-threonine and L-alanine co-doping. (**a**) Topography and (**d**) deflection; (**b**) amplitude and (**e**) phase of the vertical piezoresponse; (**c**) amplitude and (**f**) phase of the lateral piezoresponse.

The PFM measurements in pure α-glycine revealed some vertical piezoresponse without any lateral signal (Figure 1). In all doped crystals, a vertical response was also observed but all doped crystals exhibited notable lateral (shear) piezoresponses (Figure 2, Figure 3 and Figure 4). The largest shear response was found in crystals doped with L-alanine (Figure 3). However, in this case, the response was distributed inhomogeneously across the layers. The facets with high and low values for the piezoresponse were also observed in the histograms, meaning that the doping may have involved adjacent monolayers [9]. A similar distribution could be found in co-doped crystal, but with a much lower value for the piezoresponse. Interestingly, L-threonine-doped crystals revealed an almost uniform distribution of the piezo-activity within the $\left(0\bar{1}0\right)$ facets.

It should be noted that the locally measured electromechanical response is determined by two fundamental contributions: the true piezoresponse (proportional to the effective longitudinal piezocoefficient) and the electrostatic effect (Maxwell stress). In this case, the vertical force has an additional electrostatic term [14,15]:

(1) $$S_{\omega }=\frac{1}{k^{*}}C^{\prime }U_{sp}U_{AC},$$

where k* is the tip-sample contact stiffness, *C*’ is a coefficient depending on the tip-sample system geometry and electrical properties of the sample, and *U_sp_* is a surface potential. This parasitic response can be very large, especially if the true piezoelectric signal is weak [16]. However, it can have an effect on the vertical signal only because the electrostatic force is, in principle, perpendicular to the surface of the sample.

**Figure 5:**
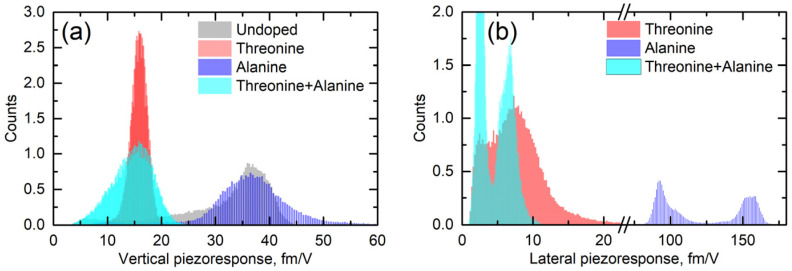
Histograms of (**a**) vertical and (**b**) lateral piezoresponses in α-glycine crystals doped with different molecules (L-alanine, L-threonine, and L-alanine + L-threonine).

Lateral (shear) piezoresponse is much more sensitive to structural distortions than the vertical one [17]. Figure 5 shows the statistical distribution of the apparent vertical and lateral responses measured across all undoped and doped crystals. It can be seen that doping with L-alanine did not have any effect on the vertical signal, but in L-threonine and co-doped samples it was strongly decreased (Figure 5a). This fact cannot be explained based on the crystal structure and was likely engendered by the electrostatics.

The lateral PFM response revealed a clear topography influence in all samples studied. However, this is not a commonly observed PFM cross-talk. Such an effect was expected because polar doping in α-glycine can be quite inhomogeneous and depends on the specific crystal tread. This statement is supported by the fact that the amplitude of the PFM response did not depend on the height values, e.g., the difference of about 10 nm in height in the top part in Figure 3 did not lead to the lateral PFM signal change, while about a 1–2 nm change of height in the square regions in the center of Figure 3 led to a significant deviation in the PFM response. The change of the lateral signal could be found also on the step edges (Figure 2c), where it correlated well with the deflection signal and was stronger in L-threonine-doped samples (Figure 2c,f) compared to L-alanine-doped ones (Figure 3c,f), as the overall level of the PFM response in threonine-doped samples was significantly lower. As such, the changes in the PFM response at the edges can be understood as a consequence of an artifact in the measurements, which gave larger impact to the samples with lower piezoelectric contributions.

Due to the uncertainties of the signal measurements at such a low-signal scale, it was difficult to analyze the values of the piezoelectric coefficients and their dependence on doping. However, some semiquantitative analysis could be performed by comparing the responses and structural aspects in the four studied groups of samples, and it offered several interesting observations.

Normally, α-glycine is non-piezoelectric. However, it can become piezoactive at the surface due to water absorbance or as a result of doping. The symmetry of the crystal reduces to *P*_21_ and then both the normal d_22_ coefficient and shear coefficients should exist. The vertical signal obtained for undoped glycine with no lateral signal must have been a consequence of an artifact related to electrostatic effects. Moreover, it implies that water from air did not induce surface piezoelectricity under the conditions of the experiment.

Extending this logic to L-threonine-doped samples, it can be concluded that the signal in these samples represents a real bulk piezoelectric effect, as both normal and shear coefficients were present and both were comparable. This agrees well with the fact that the doped samples were pyroelectric and, therefore, polar. Additionally, L-threonine samples exhibited small wavy domains not seen in the topography, which also confirmed the piezoelectric nature of the signal.

In L-alanine-doped samples, the lateral signal was highly inhomogeneous and much larger than the vertical signal. This strongly suggests that the lateral signal is definitively related to the bulk piezoelectricity and that there is a factor that enhances explicitly lateral movement at the near-surface layer probed with PFM (Figure 5b). Since water is not a suspect, it has to be concluded that, in L-alanine-doped samples, shearing of the surface layer is easier than in L-threonine-doped samples due to their smaller size. The 〈010〉 direction of glycine is a direction of cleavage. Thus, it is the easiest to shear. However, threonine has a polar group next to the methyl group, which introduces more dipole–dipole interactions. This is not conducive to the shearing of layers perpendicular to the 〈010〉 direction. This also agrees with the fact that in pure α-glycine no shear coefficient was observed because, in the absence of a true bulk piezoelectric effect, no shear strain is generated at all. These arguments are also fully consistent with the fact that, according to the modeling presented in [9], threonine induces more than 30-fold larger polarization of the host lattice than alanine, which definitely decreases the ability of the surface layers to shear. Additionally, co-doping of the crystal by alanine and threonine reduced the shear distortion in comparison to crystals doped solely with alanine (Figure 4 and Figure 5b).

In view of the considerations presented above, the behavior of the mixed doped crystals can be understood as a result of two opposing tendencies: L-threonine doping suppresses the shear of the top layers while L-alanine doping does not impede it. Since the dopant concentration is small, the formation of areas with higher and lower shear elastic compliances to the surface layer is very much possible, leading to the behavior which is an intermediate case between the L-threonine and L-alanine doping.

## 4. Conclusions

In this work, we applied a PFM method to study tiny vibrations (of the order of tens of fms) excited on the $\left(0\bar{1}0\right)$ surface of undoped and doped (<1% wt·wt^−1^ concentration) α-glycine crystals grown from aqueous solution. We showed that undoped α-glycine shows no lateral response, which is in accordance with the fact that it is not piezoelectric. L-threonine-doped samples show comparable vertical and lateral responses, which is in accordance with the fact that they are proven to be pyroelectric in the 〈010〉 direction and, therefore, polar. For L-alanine-doped samples, strong enhancement of the shear response was observed, which we attribute to the fact that shearing of the surface layer is easier in L-alanine-doped samples because alanine, in contrast to threonine, does not have a polar group in the functional part. Our data fully support the previously published theoretical modeling of local distortions induced in α-glycine by dopant incorporation.

## Data Availability

The data presented in this study are available on request from the corresponding author.
